# YAP/TEAD Co-Activator Regulated Pluripotency and Chemoresistance in Ovarian Cancer Initiated Cells

**DOI:** 10.1371/journal.pone.0109575

**Published:** 2014-11-04

**Authors:** Yan Xia, Yin-Li Zhang, Chao Yu, Ting Chang, Heng-Yu Fan

**Affiliations:** 1 Assisted Reproductive Centre, Shaanxi Maternal and Child Care Service Hospital, Xi′an, China; 2 Life Sciences Institute and Innovation Center for Cell Biology, Zhejiang University, Hangzhou, China; 3 Department of Neurology, Tangdu Hospital, the Fourth Military Medical University, Xi′an, China; Institute of Molecular and Cell Biology, Biopolis, United States of America

## Abstract

Recent evidence suggests that some solid tumors, including ovarian cancer, contain distinct populations of stem cells that are responsible for tumor initiation, growth, chemo-resistance, and recurrence. The Hippo pathway has attracted considerable attention and some investigators have focused on YAP functions for maintaining stemness and cell differentiation. In this study, we successfully isolated the ovarian cancer initiating cells (OCICs) and demonstrated YAP promoted self-renewal of ovarian cancer initiated cell (OCIC) through its downstream co-activator TEAD. YAP and TEAD families were required for maintaining the expression of specific genes that may be involved in OCICs' stemness and chemoresistance. Taken together, our data first indicate that YAP/TEAD co-activator regulated ovarian cancer initiated cell pluripotency and chemo-resistance. It proposed a new mechanism on the drug resistance in cancer stem cell that Hippo-YAP signal pathway might serve as therapeutic targets for ovarian cancer treatment in clinical.

## Introduction

Ovarian cancer is the most lethal of gynecologic malignancies, primarily due to a lack of early detection, which results in most patients being diagnosed at an advanced stage of this disease [1], [2]. The mechanisms underlying cancer drug resistance and recurrence remain uncertain. Recent evidence suggests that some solid tumors, including ovarian cancer, contain distinct populations of stem cells that are responsible for tumor initiation, growth, chemo-resistance, and recurrence [3]–[6]. There is some thought that chemotherapeutic resistance by ovarian cancer is primarily due to the existence of small populations of cancer stem cell (CSCs).

Some studies reported that CSCs organized anchorage-independent, autonomous, spherical structures [7]. Similar structures were observed in ovarian cancer patient ascites cells, which included a small subpopulation of tumor-propagating cells that were capable of organizing into spheroids. It is known that high expression levels of stem cell markers, such as OCT-4, SOX-2, Nanog, and Notch-1, can be detected in CSCs [8]. Some cell surface markers are also highly expressed by CSCs, including CD44, CD117, and CD133 [9], [10]. It is well accepted that cancer cells with high CD44 and CD117 expression become highly tumorigenic and can reestablish their original tumor hierarchy [11].

A stem cell pool that includes cancer stem cells is also tightly regulated by signaling pathways from the micro-environment of the stem cell niche. Among these, Hippo pathway has attracted considerable attention, and some investigators have focused on YAP functions for maintaining stemness and cell differentiation [12], [13]. Ectopic YAP expression prevents ES cell differentiation *in vitro* and maintains the stem cell phenotype [14], [15]. However, to date, TEAD family members, which are YAP downstream co-activators, have not been thoroughly investigated in cancer stem cells.

Recent studies showed that the interactions among several pathways, including the Hedgehog [16], Wnt [17]–[19], MAPK [20], PI3K [21], and Hippo pathways [22]–[24], were involved in stem cell pluripotency and regulating carcinogenesis. Knockdown of the Hippo pathway core components affected tissue homeostasis in the flatworm *Macrostomum lignano* and caused the hyper-proliferation of stem cells [12]. LATS2, a tumor suppressor kinase of the Hippo pathway, post-transcriptionally represses human cell reprogramming [25]. YAP is functionally important for the tumor suppressive effects on LKB1, an upstream cancer suppressor in the MAPK pathway [26].

In this study, we successfully isolated stem cell spheres from mouse tumor xenografts that were derived from human ovarian cancer cells. These sphere-forming cells were highly tumorigenic and could serially propagate with their original tumor phenotypes. Based on this enhanced, reproducible tumorigenicity, we designated these sphere-forming cells ovarian cancer initiating cells (OCICs), in accordance with previously accepted terminology. This sub-population of cancer cells also had enhanced OCICs' stemness and drug resistance through YAP/TEAD regulating the specific genes expression. These results supported recent observations, including our own, that YAP-TEADs determined ovarian cancer malignancy levels and provided additional mechanistic insights regarding the roles of YAP and TEADs in ovarian cancer.

## Materials and Methods

### Ovarian cancer initiating cell (OCIC) isolation and culture

To obtain OCICs, we subcutaneously injected cells of the ovarian cancer cell line A2780 into nude mice (2×10^6^ Cells per mouse). After a tumor diameter reached about 1.5 cm (usually at four weeks after injection), we removed the tumor tissue, cut it into small pieces, and digested it with collagenase to prepare single cell suspensions. Then the collected single cells were cultured in serum-free DMEM-F12 (Invitrogen) supplemented with 5 µg/ml of insulin (Sigma), 20 ng/ml of human recombinant epidermal growth factor (EGF; Invitrogen), 10 ng/ml of basic fibroblast growth factor (b-FGF; Invitrogen), and 0.4% bovine serum albumin (BSA; Sigma) in Ultra Low Attachment plates (Corning). OCICs and the control cells were all separated from other cells using continuous density gradient centrifugation. The control cells were also obtained by injecting A2780 cells into nude mice and the separation methods were similar to those used for OCICs. They were cultured in Ultra Low Attachment plates (Corning) and the medium was similar with OCICs except that the culture medium included 10% fetal bovine serum (FBS). All media included penicillin (10 units/ml) and streptomycin (10 ng/ml) and cells were grown in a humidified incubator at 37°C with 5% CO_2_. Medium was changed every 3 days.

### Nude mouse xenograft model

Mice were treated in accordance with the NIH Guide for the Care and Use of Laboratory Animals, approved by ethics committee of Zhejiang University. Mice were housed in a temperature-controlled room with proper darkness-light cycles, fed with a regular diet, and maintained under the care of the Laboratory Animal Unit, Zhejiang University, China. The ovarian cancer cell-transplanted nude mice were injected 2×10^6^ cells and examined daily for about three weeks. After the tumor diameter reached to 1.5 cm, the mice were euthanized using CO_2_ inhalation method before being sacrificed. To assess OCIC tumorigenic capability *in vivo*, spheroids were counted, resuspended in 100 µl of PBS, and then subcutaneously injected into the left flanks of 4-week-old female nude mice. Cell concentrations used ranged from 10^4^ to 10^5^ in OCIC and control groups and the mice were divided into two groups and four mice in each subgroup. Starting at one week after injection, engrafted mice were observed daily for tumor masses and volume. A mouse was humanely sacrificed when a tumor diameter reached 1.5 cm. Xenograft tumors were dissected out, fixed in 4% paraformaldehyde, embedded in paraffin, and sectioned with a Leica rotary microtome (5 µm thickness). Sections were used for H&E staining, immunohistochemistry, and histological assessments.

### Immunofluorescence staining

OCIC spheroids were harvested and placed on glass slides, fixed in paraformaldehyde (4°C, 30 min), permeabilized with PBS that contained 0.3% Triton X-100 (PBST), and incubated with blocking buffer (PBST containing 5% bovine serum albumin). Spheroids were sequentially probed with the primary antibodies and Alexa Fluor 594- or 488-conjugated secondary antibodies (Molecular Probes). Slides were mounted using VectaShield with 4′, 6-diamidino-2-phenylindole (DAPI, Vector Laboratories). Digital images were acquired using an epifluorescence microscope (Nikon Eclipse 80i) with 4-100X objectives.

### Immunohistochemistry

Tumor xenografts were fixed overnight in 10% PBS buffered 4% paraformaldehyde, and then embedded in paraffin. Sections were cut with a Leica RM2235 microtome at 5 µm thickness and stained with the indicated primary antibodies for immunohistochemistry using a Vector ABC kit (Vector Laboratories). Briefly, sections were deparaffinized, rehydrated, and incubated in 0.3% H_2_O_2_. After antigen retrieval using 10 mM sodium citrate (pH 6.0), sections were incubated in normal goat serum. These samples were then probed with primary antibodies. After washing with PBS that contained 0.05% Tween-20 (PBS-T), samples were incubated with a secondary antibody and washed again with PBS-T before incubation with ABC solution. Color was developed with diaminobenzidine (DAB) substrate (DAB Substrate Kit, Vector Laboratories). Sections were washed, counterstained, dehydrated, and mounted with Vectamount permanent mounting medium (Vector Laboratories). Sections were observed under a Nikon Eclipse 80i Microscope (Nikon Corporation). The negative control was replaced the primary antibody with PBS incubation.

### Lentiviral shRNA infection and clone formation assay

Lentivirus that encoded for *Yap* and *Tead1/3/4* short hairpin RNAs (shRNAs) or non-target oligonucleotides as a control were from GenePharma (Shanghai, China). Short interfering RNAs for *Yap* and *Tead1/3/4* selected from different target sequences were inserted into LV-3/GFP + Puro vectors. Cells in spheres were infected with *Yap* and/or *Tead1/3/4* shRNA lentivirus for 48 h. *Yap*-specific shRNAs were: 5′-CCCAGTTAAATGTTCACCAAT-3′ (shYAP#1) and 5′-GCCACCAAGCTAGATAAAGAA-3′ (shYAP#2). Two shRNAs were used to target *Tead1*, *Tead3*, and *Tead4* together: 5′-ATGATCAACTTCATCCACAAG-3′ and 5′-GATCAACTTCATCCACAAGCT-3′. Interference efficiency was verified by RT-PCR and Western blotting. Lentivirus infected OCICs were plated in triplicate in 12-well plates (1,000 cells/well) for 14 days. Medium was replaced every 48 hours and visible colonies were counted by light microscopy.

### Spheroid differentiation assessments

Cell spheres were cultured under standard differentiating conditions (DMEM/F12 supplemented with 10% FBS) and in Ultra Low Attachment plates (Corning). After 14 days in culture, cell morphology was assessed using a Zeiss Axiovert 40 inverted microscope with Axio-Vision software. Cell surface markers (CD44 and CD117) were detected by immunofluorescence staining. An epithelium differentiation marker, Cytokeratin-7 (CK-7), and ovarian cancer antigen-125 (CA125) were used for immunofluorescence staining, followed by incubation with a FITC-labeled goat anti-mouse or anti-rabbit IgG. Nuclei were counterstained with 4, 6-diamidino-2-phenylindole (DAPI; Santa Cruz).

### Chemotherapy agent sensitivity assays

Cell spheroids were dissociated and seeded at 4,000 cells/well in 96-well Ultra Low Attachment plate (Corning) and cultured with serum-free DMEM/F12 supplemented with growth factors. OCICs and control ovarian cancer cells were treated with cisplatin (20 to 60 µM), taxol (2 to 20 µM), or bleomycin (20 µM to100 µM) for 48 h. After culture for 48 h, cell viability was assessed using a cell counting kit-8 (Dojndo, Japan), according to the manufacturer's instructions. Cell survival rates were defined as the percentage of surviving drug-treated cells divided by untreated control cells. All experiments were performed in triplicate.

### Quantitative PCR microarray analysis and real-time RT-PCR

Human Cancer Drug Resistance RT^2^ Profiler PCR array (QIAGEN #330231) was used to determine the expression profiles of 84 genes involved in the chemotherapy regulation. Primers for 84 test genes and 5 housekeeping genes (*B2m*, *Hprt1*, *Rpl13a*, *Gapdh*, and *Actb*) were included on each 96-well plate. A First Strand kit (QIAGEN #330401) and SYBR Green qPCR Mastermix (QIAGEN #330500) were from SABiosciences. qPCR was performed according to the manufacturer's instructions (SABiosciences RT^2^ Profiler PCR Array System) using a Bio-Rad CFX96TM Real-Time PCR device. Data analysis was done using the online SABiosciences PCR Array data analysis, as described in the manufacturer's protocol.

Spheroid cells, differentiated spheroid cells, or primary tumor cells were placed in RNAase-free microtubes. Total RNA extraction and reverse transcription were done with RNAiso Plus and a Reverse Transcription Mix kit (Takala), according to the manufacturer's instructions. Total RNA (0.5 µg) was reversed transcribed using a cDNA Synthesis Kit (Bio-Rad). Real-time PCR reactions were run using a CFX96 Real-Time PCR Detection System (Bio-Rad). Each cDNA sample was tested in triplicate. To quantify gene expression changes, the △△Ct method was used to calculate relative fold-changes after normalizing to *Actb* (β-actin) mRNA levels. The gene-specific RT-PCR primer sequences are shown in Table S1.

### Protein extraction and Western blotting analysis

Cells were lysed with cell lysis buffer (Beyotime) that included an appropriate volume of a protease inhibitor cocktail (Roche). Total proteins were separated by SDS-PAGE and then transferred to polyvinylidene fluoride membrane (Milipore, USA). Non-specific binding sites were blocked with 5% skim milk in TBST at room temperature for 1 h. After probing with primary antibodies, membranes were incubated with horseradish peroxidase-linked anti-rabbit antibodies (Cell Signaling Technologies, Danvers, MA) and then washed. Bound antibodies were visualized using an Enhanced Chemiluminescence Detection Kit (Amersham). The primary antibodies were: anti-YAP, anti-OCT-4, anti-Notch-1, anti-actin, all of which were from Cell Signaling Technology. An anti-TEAD1 (1∶1000; 5178-1) antibody was from Epitomics. Anti-TEAD2 (1∶1000; LS-C119063) and anti-TEAD3 (1∶1000; LS-C30406) antibodies were from Lifespan Biosciences. Anti-TEAD4 (1∶1000; ab97460) antibody was from Abcam. Proteins were visualized using a Dura Super Signal Substrate (Millipore, USA).

### Statistical analysis

All assays were done in triplicate. GraphPad Prism software (GraphPad Prism, San Diego, CA) was used to compare group results by Chi-square tests or ANOVA, as appropriate. Differences were considered significant for p<0.05.

## Results

### Ovarian cancer initiated spheres have cancer stem cell properties

**Figure 1:**
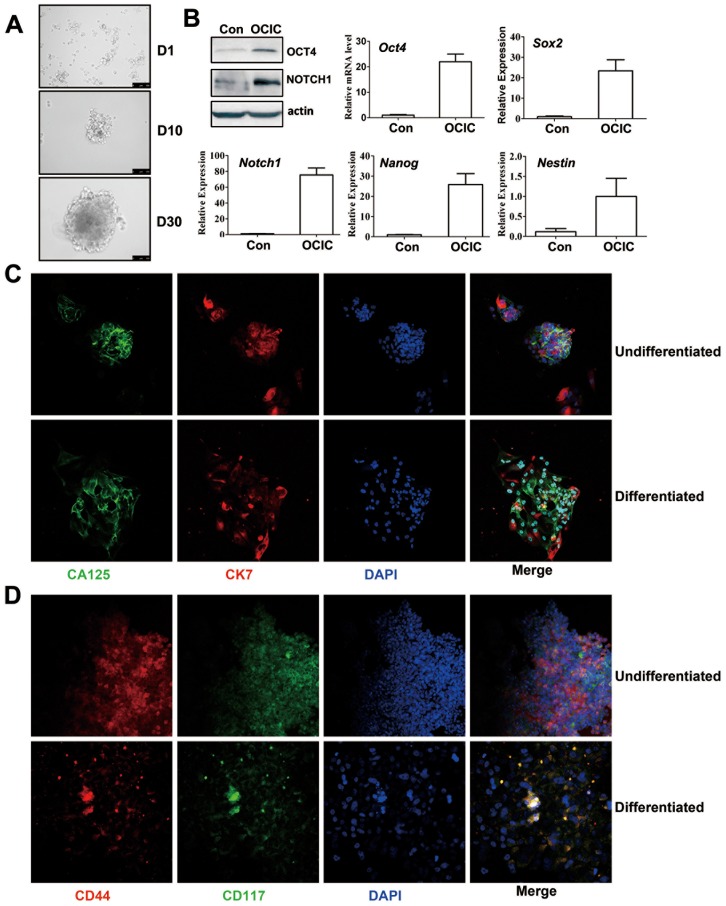
Isolation and culture of ovarian cancer-initiating cell (OCIC) spheroids with characteristics of self-renewal and anchorage-independent growth. **A**: Images of non-adherent spherical cell clusters derived from cultured primary ovarian cancer cells. Scale bars  = 100 µM. **B**: Western blotting and real-time RT-PCR results showing enhanced expression of the indicated genes in OCICs. Relative mRNA levels were determined by normalizing to endogenous β-actin mRNA levels (used as an internal control) using Microsoft EXCEL. For each indicated gene, the relative transcript level of the control sample (left-hand bar of each graph) was set at 1. The relative transcript levels of other samples were compared to the control, and fold-changes are shown in the graph. **C–D**: Representative immunofluorescence staining results for CA125, CK7, CD44, and CD117 expression in undifferentiated and differentiated OCICs. Nuclei were stained with DAPI. Scale bar  = 100 µM for all panels.

Primary ovarian cancer cells were dissociated and placed in uncoated culture flasks in serum-free medium supplemented with EGF, b-FGF, insulin, and BSA. After one week in culture, non-adherent spherical clusters of cells were observed at the bottom of these flasks. At one month later, spheres with increased sizes were clearly observed (Fig. 1A). Usually 5×10^6^∼1×10^7^ digested xenograft cells were put into culture, and approximately 2–4% of them were converted to OCICs under the serum deprived conditions. Real-time RT-PCR and Western blotting results showed that the stem cell markers OCT-4, SOX-2, Nestin, Notch-1, and Nanog were all highly expressed by these cells in spheres (Fig. 1B).

Next, we investigated if these sphere cells had anchorage-independent self-renewal. We cultured spheroids for 14 days under differentiating conditions (growth factors withdrawn and medium that contained 10% FBS). The spheroid cells changed from floating cells to adherent cells, acquired an epithelial morphology, and formed CA125 and CK-7-positive symmetric colonies (Fig. 1C). (CA125 and CK-7 are well established cell surface markers of differentiated epithelial ovarian cancer cells.) In addition, the previously reported cell surface markers of ovarian epithelial stem cells, CD44 and CD117, were expressed at much higher levels in sphere cells than in differentiated flat cells (Fig.1D).

### Sphere-forming cells are strongly tumorigenic

**Figure 2:**
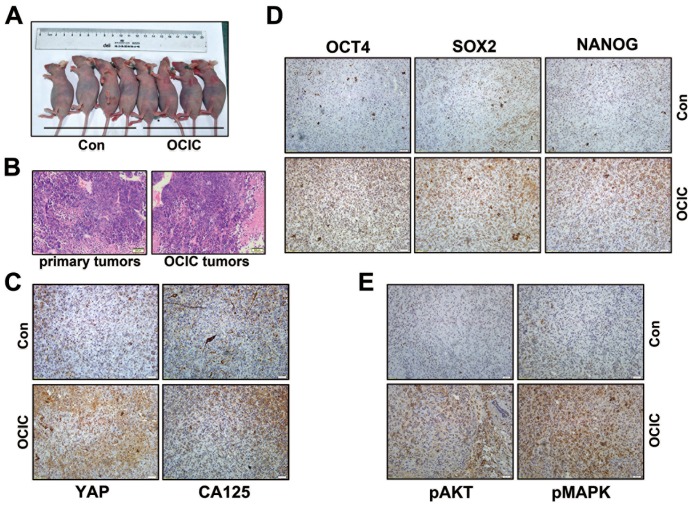
OCICs have stronger tumorigenic capability than primary ovarian cancer cells. **A**: Images of nude mice showing xenograft ovarian tumor formation after injecting OCIC spheres. Cell concentrations used 10^4^ in each group. **B**: H&E staining results showing the histology of tumors derived from subcutaneously transplanted OCIC spheroids. **C**: Representative IHC results for CA125 and YAP expression in human xenograft tumors derived from OCICs. Specific protein expression is indicated by the brown color and nuclei (blue) were counterstained with DAPI. **D**: Representative IHC results for the expression of the pluripotency markers OCT4, SOX2, and NANOG in xenograft ovarian tumors derived from OCIC spheroids. **E**: Representative IHC results for phosphorylated AKT and pMAPK (ERK1/2) expression in the xenograft tumors derived from OCICs. Scale bar  = 200 µM for B-E panels.

We examined whether these identified sphere-forming cells were as strongly tumorigenic as other reported epithelial cancer initiating cells. Comparable numbers of sphere cells and control cells were subcutaneously injected into the flanks of nude mice. At two weeks later, tumors were found in three of four athymic nude mice that had been injected with 10^4^ sphere cells. However, tumors were not found in mice that had been injected with differentiated ovarian tumor cells, even at 10^5^ cells per engraftment (Fig. 2A). Cell concentration shown in Fig. 2A is 10^4^ per mice and other concentrations results have not been shown.

H&E staining results showed that the tumors derived from the injected sphere-forming cells had a histological similar to that of A2780 cell injected tumors (Fig. 2B). These tumor tissues expressed high levels of YAP and were positive for CA-125, an ovarian adenocarcinoma marker (Fig. 2C). These results showed that the sphere-forming cells we had identified were highly tumorigenic and could serially propagate to their original tumor phenotype. Based on this enhanced, reproducible tumorigenicity, we designated these sphere-forming cells ovarian cancer initiating cells (OCICs), in accordance with previously accepted terminology. In addition, we also detected the stem cell markers (OCT4, SOX2, and Nanog) expression and AKT/MAPK phosphorylation levels in these tumor tissues with different cell origin (Fig. 2D–E). The results also showed that these genes have a higher expression levels than that in the controls.

### YAP and TEAD are required for maintaining OCIC pluripotency

**Figure 3:**
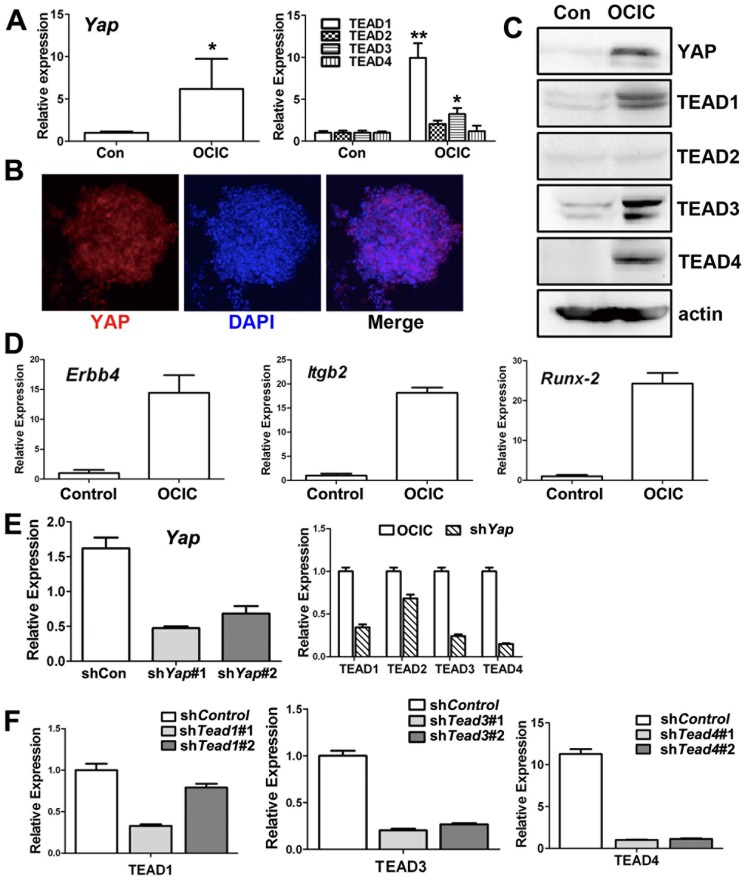
YAP and TEAD are required for maintaining OCIC pluripotency. **A-C**: Real-time RT-PCR (A), immunofluorescence staining (B), and Western blotting (C) results for YAP and TEAD1-4 expression levels in primary ovarian cancer cells (control) and OCICs. Nuclei were stained with DAPI. **D**: Real-time RT-PCR results for mRNA levels of known YAP/TEAD target genes, including *Runx2*, *Itgb2*, and *Erbb4*, in primary ovarian cancer cells (control) and OCIC cells. **E-F**: Real-time RT-PCR results for the RNAi depletion efficiencies of YAP, TEAD1, TEAD3, and TEAD4, after using two different shRNAs for each gene.

A previous report showed that YAP was involved in iPS cell maintenance under undifferentiated conditions [14]. Thus, we postulated that YAP could also maintain ovarian cancer stem cell pluripotency. YAP and TEAD family members, except for TEAD2, were all expressed at significantly higher levels by OCICs than by differentiated ovarian cancer cells (Fig. 3A–C). In addition, mRNA levels of known YAP/TEAD target genes, including *Runx2*, *Itgb2*, and *Erbb4*, were all significantly increased in OCIC cells, suggesting that YAP/TEAD activity was high in these cells (Fig. 3D). These results were consistent with those in a previous report [27].

**Figure 4:**
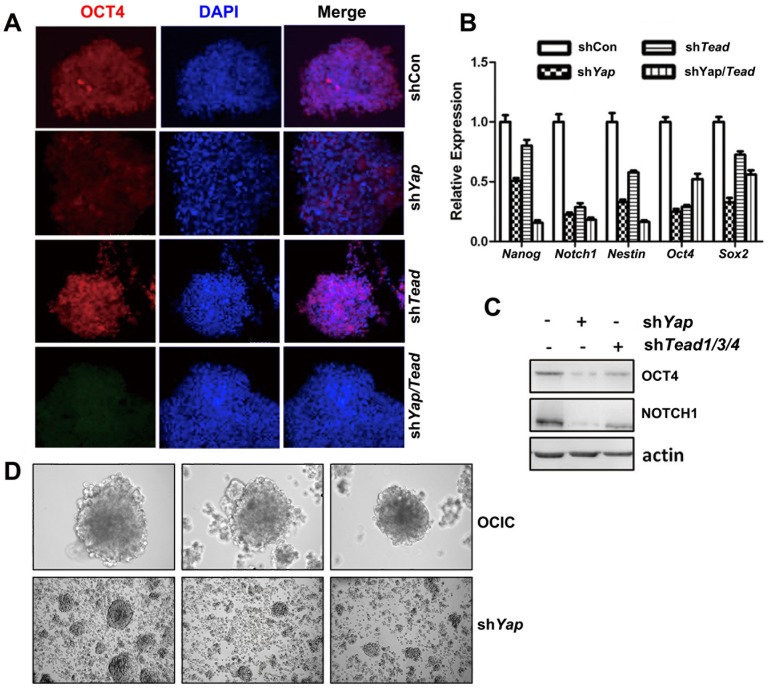
Pluripotency markers' expression in YAP- and TEAD1/3/4-silenced OCICs. **A**: Representative immunofluorescence staining results for OCT-4 expression in *Yap*- and *Tead1/3/4*-silenced OCICs. **B-C**: Real-time RT-PCR (B) and Western blotting (C) results showing that the indicated genes were down-regulated in OCICs after RNAi depletion of *Yap* and *Tead1/3/4*. **D**: Images of OCIC spherical clusters without (upper panels) and with (lower panels) sh*Yap*-treatment by 200 times magnification.

To investigate if YAP and TEAD family members were involved in maintaining OCIC pluripotency, we knocked-down *Yap* and *Tead1/3/4* expression in OCICs by using shRNAs. RT-PCR results confirmed the knock-down efficiencies of the indicated shRNAs (Fig. 3E–F). Immunofluorescence staining results showed that OCT-4 expression was weaker in shYAP and shTEAD treated OCICs than in control cells (Fig. 4A). In addition to OCT-4, other stem cell markers' expression was also decreased, as determined by RT-PCR and Western blotting (Fig. 4B–C). In a clony formation assay, when sphere-forming cells were treated with *Yap* shRNA, the spheroids were smaller (Fig. 4D). Similar morphological changes were also observed for sh*Tead1/3/4*-treated OCICs (data not shown). These results showed that a YAP/TEAD co-activator was required for maintaining OCIC pluripotency.

### YAP-TEAD confers chemotherapeutic drug resistance to OCICs

**Figure 5:**
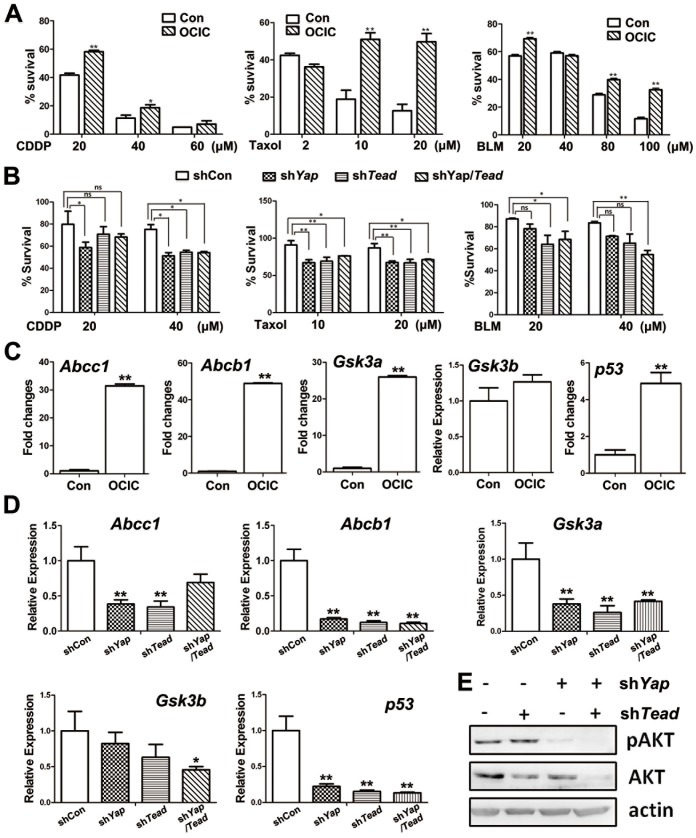
YAP-TEAD confers chemotherapeutic drug resistance to OCICs by regulating specific target genes' expression. **A.** Survival rates of primary ovarian cancer cells (control) and OCICs after treatment with cisplatin (CDDP), taxol, or bleomycin at the indicated concentrations. OCICs and control cells were treated with drugs for 48 h. *, P<0.01, compared with the corresponding control group. **, P<0.001, compared with the corresponding control group. **B.** Survival rates of OCICs with or without *Yap* and *Tead1/3/4* knockdown after treatment with CDDP, taxol, or bleomycin at the indicated concentrations for 48 h. ns, not significant; *, P<0.01; **, P<0.001. **C-D**: Real-time RT-PCR results showing that the indicated genes were expressed in OCICs at higher levels than in primary ovarian cancer cells (C). Indicated genes' expression levels in OCICs were significantly down-regulated with *Yap* and *Tead1/3/4* RNAi (D). *, P<0.01; **, P<0.001. **E.** Western blotting results for AKT and pAKT levels in OCICs with or without *Yap/Tead1/3/4* shRNA treatment.

One property of cancer stem cells is their resistance to conventional chemotherapy agents. Thus, we determined OCICs' sensitivity to cisplatin, taxol, and bleomycin treatments. Primary tumor cells exhibited sensitivities to these chemotherapeutic drugs in a dose-dependent manner (Fig. 5A). Compared with primary tumor cells, OCICs exhibited enhanced resistance to cisplatin, taxol, and bleomycin (Fig. 5A). However, OCICs with *Yap* and *Tead1/3/4* knockdown had decreased survival rates (Fig. 5B). These results supported the hypothesis that YAP-TEAD enhanced the drug resistance of the stem-like cells in ovarian cancer.

### YAP up-regulates GSK3A and ABCB1 expression to enhance OCICs' drug resistance

To determine the drug-resistant genes involved in regulating OCICs, we used drug-resistant gene microarray analysis for OCICs and identified multiple putative chemoresistance-related genes that were highly expressed in OCICs. A tabular synopsis of their microarray findings with the respective fold changes was provided in Table S1. The drug-resistance-related genes ABCB1, ABCC1 GSK3A, had significantly higher expression levels in OCICs than in primary ovarian cancer cells (Fig. 5C). We further investigated if these genes were regulated by YAP-TEADs in OCICs. Among these genes, ABCB1 and GSK3A were significantly down-regulated in *Yap/Tead*-depleted OCICs, whereas ABCC1 expression was not significantly affected (Fig. 5D).

In addition, *Gsk3a*, *Gsk3b,* and *p53* gene expression was also detected in OCICs (Fig. 5C). Among these, *Gsk3a* and *p53* were remarkably down-regulated in *Yap/Tead*-depleted OCICs and *Gsk3b* changed little (Fig. 5D). These results suggested that YAP and TEADs enhanced OCICs' drug resistance by up- and down-regulating those genes involved in drug metabolism and cell survival.

The PI3K signaling pathway is known to cooperate with the Hippo pathway to regulate cell growth and proliferation [28]. Phosphorylated AKT levels were high in OCICs and decreased with *Yap* and/or *Tead1/3/4* depletion. Interestingly, the total AKT levels were also downregulated in *Yap/Tead1/3/4* co-depleted OCICs. These results also demonstrated that YAP/TEADs were required for maintaining PI3K/AKT pathway activity in OCICs (Fig. 5E).

### MAPK pathway genes are involved in YAP maintained OCIC pluripotency

**Figure 6:**
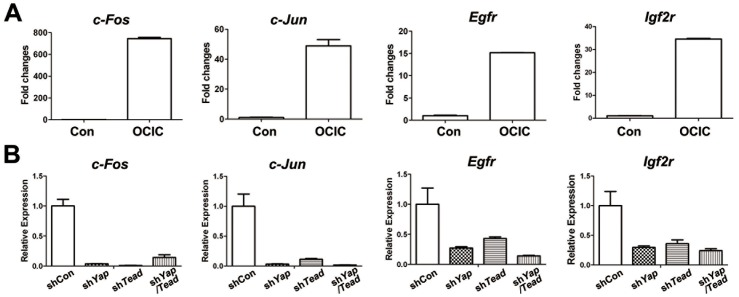
MAPK pathway genes regulated by YAP in OCICs. **A.** Real-time RT-PCR results showing that the indicated genes were expressed in OCICs at higher levels than in primary ovarian cancer cells (control). **B.** Real-time RT-PCR results showing that the indicated genes' expression levels were significantly down-regulated in OCICs with *Yap*/*Tead1/3/4* RNAi.

The results of qPCR microarray analysis (Table S2) showed that several genes involved in MAPK pathways, including *c-Fos*, *Egfr*, and *Igf2r*, had higher expression levels in OCICs than in primary ovarian cancer cells (Fig. 6A). Their expression was down-regulated after *Yap* and *Tead1/3/4* shRNA treatment (Fig. 6B). Because C-JUN and C-FOS form the AP-1 complex and function together, we examined *c-Jun* gene expression in these samples and found similar results (Fig. 6A–B). The *Egfr* and *Igf2r* genes encode for the epidermal growth factor receptor (EGFR) and the insulin-like growth factor 2 receptor (IGF2R), respectively. These are important cell membrane receptors that transmit key extracellular signals to the nucleus by activating MAP kinase cascades. Phosphorylated and activated MAP kinases translocate from the cytoplasm to the nucleus to mediate C-FOS and C-JUN transcription activation. Some studies showed that YAP regulated TEAD genes to up-regulate the *Areg* gene, which interacts with the EGF/TGF-alpha receptor to promote the growth of normal epithelial cells [29], [30]. Thus, our results suggested that these genes in MAPK pathways may be involved in YAP maintained OCIC pluripotency.

## Discussion

Tumor occurrence and progression are closely related to abnormal somatic cell development and differentiation. There are small numbers of specific cells or so-called stem cell in our body included ovarian tissue that are capable of self-renewal and directional differentiation [31]–[33]. As a part of these cells, cancer stem cells have recently attracted attention. A previous study showed that a much smaller cancer stem cell population indeed existed in some cancer tissues, which initiated the development of cancer cells and was related to stem cell property maintenance, tumorigenesis, malignant metastasis, and recurrence [34]. Cancer stem cells have several important features, included clone formation capability, the expression of stem cell markers and specific cell surface markers, cell differentiation and morphological identification, and strong tumorigenic capacity.

In this study, we successfully isolated ovarian cancer stem-like cells from tumor-bearing mice and demonstrated that these cells had the cancer stem cell characteristics described above. After about one month in culture, OCICs formed clusters that contained numerous cells and had diameters up to 500 µm. OCICs not only had strong clonogenic ability but also expressed cancer stem cell markers at high levels. Cell differentiation results showed that the spheroids we isolated indeed had the characteristics of epithelial ovarian cancer. The tumorigenic rates of these OCICs were significantly higher than those of primary ovarian cancer cells and this data has not been shown in Figure 2.

Many signaling pathways, such as the Wnt, PI3K, MAPK signaling pathways, have been reported to be closely associated with stem cells and the Hippo pathway [18], [19]. The Hippo pathway has also received considerable attention with regard to stem cell regulation and tumorigenesis mechanisms [23], [35], [36]. Some studies show that the YAP gene regulated epithelial stem cell repair and intestinal stem cell and hematopoietic stem cell function [37]–[40]. Tumor-propagating cells and activity contributed to lung tumor progression and metastasis in CD24-dependent and Yap/Taz-dependent pathways [41]. YAP was inhibited by catenin during epidermal stem cell proliferation and skin cancer development via the Wnt signal pathway [42]. In contrast, YAP restricted Wnt signals during intestinal regeneration. In our study, AKT and MAPK phosphorylation levels were dramatically higher expression not only in OCIC tumor tissues but also in OCICs.

Transgenic expression of YAP reduced Wnt target gene expression and resulted in the rapid loss of intestinal crypts. In addition, a loss of YAP resulted in hyperplasia and the expansion of intestinal stem cells (ISCs) and niche cells [38]. YAP maintained undifferentiated embryonic stem (ES) cells and YAP levels were increased during induced pluripotent stem (iPS) cell reprogramming [14]. However, it is unknown if YAP/TEAD are involved in cancer stem cell development, maintenance, and differentiation.

Our current study is the first to report that a YAP regulation mechanism was involved in ovarian cancer stem cells. Our results showed that YAP maintained undifferentiated OCICs and that differentiated OCICs' expressions of stem cell markers were down-regulated with YAP knockdown. YAP downstream factors, TEADs, were also high expressed in OCICs; however, with YAP knockdown, their expressions declined dramatically. In OCICs, TEAD1/3/4 expression was at a high level, whereas TEAD2 expression was at a low level among these four subtypes. This result was similar to those in a previous study [43]. These results suggested that YAP and TEAD members were all co-activated in cancer stem cells and that their coordinated interactions may have a key role in cancer stem cell pluoripotency, self-renewal regulation.

Based on the recent development of new chemotherapy drugs, tumor cure rates have increased substantially, although the bottleneck problem of tumor drug resistance remains unresolved. The existence of cancer stem cells might be a major cause of tumor chemotherapy failure and recurrence [3]. In our study, OCICs were treated with different concentrations of three drugs (cisplatin, taxol, and bleomycin) commonly used for clinical ovarian cancer therapy. Our results showed that OCICs were significantly more resistant to these drugs than were primary ovarian cancer cells. More importantly, YAP/TEAD activities were required to maintain OCIC drug resistance. These results suggest that modulating YAP and TEAD activities may be an effective strategy to prevent the recurrence and chemoresistance of ovarian cancers.

YAP interacts with other signaling pathways to form a network to regulate cell signal transduction. In our study, several important signaling pathways were also involved in Yap-regulated OCIC maintenance. These extracellular signals might have stimulated two receptor genes, EGFR and IGF2R, and activated RAS-MAPK cascades, which further upregulated the expression of AP1 transcription factors, C-JUN and C-FOS. Our study results also suggested that the PI3K pathway might have synergetic effects with YAP/TEADs in OCIC maintenance. In addition, the OCICs generated in this study had significant high P53 levels than the primary ovarian cancer cells. Literature suggests that p53 dysfunction can enhance the self-renewal ability of ovarian stem-like tumor cells. However, previous studies showed that in ovarian cancer cells, the highly expressed P53 were often mutated forms without tumor repressing activity. The OCICs we generated in this study were derived from xenograft formed by A2780 ovarian cancer cells. P53 was known to be mutated in this cell line [44], [45]. So the OCICs in this study may also have functional inactive P53.

Phosphorylated AKT is an important regulator of several cellular processes. AKT phosphorylation was detected in OCICs and decreased when YAP/TEADs were silenced. The decrease in total and phosphor-AKT levels in YAP/TEAD-depleted ICOCs was accompanied by decreased OCIC survival rates and chemotherapeutic drug resistance.

In conclusion, the study reports interesting findings that may provide insights in the phenomenon of drug resistance in ovarian cancers. YAP interacted with many stem cell regulation pathways and the exact regulation mechanisms between them will be our focus in subsequent studies.

## Supporting Information

Table S1 Sequences of primers used for quantitative RT-PCR.
(DOCX)

Table S2 Results of qPCR microarray analysis.
(DOCX)
