# Supplementary material for: YAP/TEAD Co-Activator Regulated Pluripotency and Chemoresistance in Ovarian Cancer Initiated Cells
Source: PLoS One. 2014 Nov 4;9(11):e109575. doi: 10.1371/journal.pone.0109575 (PMC4219672; doi:10.1371/journal.pone.0109575)
Supplement: Table S1 — Sequences of primers used for quantitative RT-PCR. (DOCX) [file pone.0109575.s001.docx]

**Table S1.** Sequences of primers used for quantitative RT-PCR.

| **Gene** | **Primer sequences** |
| --- | --- |
| ***Notch1*** | F: AATGTGGATGCCGCAGTTG  R: ATCCGTGATGTCCCGGTTG |
| ***Oct4*** | F: GTGCCGTGAAGCTGGAGAA  R: TGGTCGTTTGGCTGAATACCTT |
| ***Sox2*** | F: GTGAGCGCCCTGCAGTACAA  R: GCGAGTAGGACATGCTGTAGGTG |
| ***Nestin*** | F: CTCCAAGAATGGAGGCTGTAGGAA  R: CCTATGAGATGGAGCAGGCAAGA |
| ***Nanog*** | F: CAACATCCTGAACCTCAGCTACAA  R: GGCATCCCTGGTGGTAGGAA |
| ***Tead1*** | F： GCTCCATTGGCACAACCAAG  R：ATGCCCAATGTGCACGAAGA |
| ***Tead2*** | F：TGCCTGAGCGATACATGATGAAC  R：CCTGACCAGGCGGTAAATGTG |
| ***Tead3*** | F：CTGTCAGACGAGGGCAAGATGTA  R：GAGCTAGAACCTGTATGTGGCTGGA |
| ***Tead4*** | F：CCAAGCTCTGGATGTTGGAGTTC  R： GATGTCCACGGCTTCGAGGTA |
| ***Yap*** | F：TAGCCCTGCGTAGCCAGTTACC  R：GCTGCTCATGCTTAGTCCACTGTC |
| ***Actin*** | F：TGGCACCCAGCACAATGAA  R：CTAAGTCATAGTCCGCCTAGAAGCA |
| ***Abcb1*** | F: TGACTCAGGAGCAGAAGTTTGAACA  R: AAATACATCATTGCCTGGGTGAAG |
| ***Abcc1*** | F: GTGATGGCGATGAAGACCAAGA  R: GCCAGCTCCCAGGCATAAAG |
| ***c-Fos*** | F: TCTTACTACCACTCACCCGCAGAC  R: GGAATGAAGTTGGCACTGGAGAC |
| ***c-Jun*** | F: CACGTGAAGTGACGGACTGTTCTA  R: CAGGGTCATGCTCTGTTTCAGG |
| ***Gsk3a*** | F: AGGCCAAGTTGACCATCCCTATC  R: AGCAGTGTCAGGGTCCACCA |
| ***Gsk3b*** | F: TGATGAATTACGGGACCCAAATG  R: GGTAGCCAGAGGTGGATTACTTGAC |
| ***Egfr*** | F: GTGGCGGGACATAGTCAGCA  R: CCCATTGGGACAGCTTGGA |
| ***Igf2r*** | F: TACAACTTCCGGTGGTACACCA  R: CATGGCATACCAGTTTCCTCCA |
| ***P53*** | F：TCAGCATCTTATCCGAGTGGAA  R：TGTAGTGGATGGTGGTACAGTCA |

F: forward primer; R: reverse primer
