# Supplementary material for: YAP/TEAD Co-Activator Regulated Pluripotency and Chemoresistance in Ovarian Cancer Initiated Cells
Source: PLoS One. 2014 Nov 4;9(11):e109575. doi: 10.1371/journal.pone.0109575 (PMC4219672; doi:10.1371/journal.pone.0109575)
Supplement: Table S2 — Results of qPCR microarray analysis. (DOCX) [file pone.0109575.s002.docx]

**Table S2.** Results of qPCR microarray analysis.

| **Location** | **Gene** | **Fold Change** |
| --- | --- | --- |
| A01 | *Abcb1* | 48.8644 |
| A02 | *Abcc1* | 31.4816 |
| A03 | *Abcc2* | 2.9006 |
| A04 | *Abcc3* | 1.2209 |
| A05 | *Abcc5* | 1.8842 |
| A06 | *Abcg2* | 2.1221 |
| A07 | *Ahr* | 3.1159 |
| A08 | *Ap1s1* | 5.3571 |
| A09 | *Apc* | 0.2966 |
| A10 | *Ar* | 1.7207 |
| A11 | *Arnt* | 0.8817 |
| A12 | *Atm* | 0.3443 |
| B01 | *Bax* | 1.8987 |
| B02 | *Bcl2* | 0.2941 |
| B03 | *Bcl2l1* | 2.0344 |
| B04 | *Blmh* | 0.0668 |
| B05 | *Brca1* | 1.0882 |
| B06 | *Brca2* | 1.1451 |
| B07 | *Ccnd1* | 2.8946 |
| B08 | *Ccne1* | 0.9731 |
| B09 | *Cdk2* | 2.567 |
| B10 | *Cdk4* | 0.7066 |
| B11 | *Cdkn1a* | 0.8818 |
| B12 | *Cdkn1b* | 5.7869 |
| C01 | *Cdkn2a* | 0.0731 |
| C02 | *Cdkn2d* | 0.7265 |
| C03 | *Clptm1l* | 1.8496 |
| C04 | *Cyp1a1* | 0.7574 |
| C05 | *Cyp1a2* | 0.2559 |
| C06 | *Cyp2b6* | 0.4722 |
| C07 | *Cyp2c19* | 1.7418 |
| C08 | *Cyp2c8* | 0.7793 |
| C09 | *Cyp2c9* | 0.2904 |
| C10 | *Cyp2d6* | 0.6346 |
| C11 | *Cyp2e1* | 1.6111 |
| C12 | *Cyp3a4* | 1.0876 |
| D01 | *Cyp3a5* | 2.962 |
| D02 | *Dhfr* | 0.1445 |
| D03 | *Egfr* | 15.1611 |
| D04 | *Elk1* | 3.9722 |
| D05 | *Ephx1* | 0.3461 |
| D06 | *Erbb2* | 2.7192 |
| D07 | *Erbb3* | 1.6239 |
| D08 | *Erbb4* | 1.9944 |
| D09 | *Ercc3* | 7.0478 |
| D10 | *Esr1* | 0.6395 |
| D11 | *Esr2* | 1.5251 |
| D12 | *Fgf2* | 0.2568 |
| E01 | *Fos* | 745.0141 |
| E02 | *Gsk3a* | 25.977 |
| E03 | *Gstp1* | 0.1248 |
| E04 | *Hif1a* | 0.7183 |
| E05 | *Igf1r* | 0.7638 |
| E06 | *Igf2r* | 34.5829 |
| E07 | *Met* | 0.208 |
| E08 | *Msh2* | 0.1616 |
| E09 | *Mvp* | 0.1046 |
| E10 | *Myc* | 0.7533 |
| E11 | *Nat2* | 0.5562 |
| E12 | *Nfkb1* | 1.9264 |
| F01 | *Nfkb2* | 2.8333 |
| F02 | *Nfkbib* | 0.3964 |
| F03 | *Nfkbie* | 0.3329 |
| F04 | *Ppara* | 0.853 |
| F05 | *Ppard* | 11.4902 |
| F06 | *Pparg* | 0.2025 |
| F07 | *Rara* | 4.5931 |
| F08 | *Rarb* | 1.6151 |
| F09 | *Rarg* | 3.0674 |
| F10 | *Rb1* | 0.8597 |
| F11 | *Relb* | 27.6022 |
| F12 | *Rxra* | 1.8303 |
| G01 | *Rxrb* | 10.795 |
| G02 | *Sod1* | 0.1517 |
| G03 | *Sult1e1* | 0.35 |
| G04 | *Tnfrsf11a* | 0.2508 |
| G05 | *Top1* | 8.597 |
| G06 | *Top2a* | 0.7183 |
| G07 | *Top2b* | 0.7421 |
| G08 | *Tp53* | 4.8806 |
| G09 | *Tpmt* | 0.053 |
| G10 | *Ugcg* | 0.3482 |
| G11 | *Xpa* | 8.4747 |
| G12 | *Xpc* | 0.8079 |
| H01 | *Actb* | 1.2007 |
| H02 | *B2m* | 0.0926 |
| H03 | *Gapdh* | 1 |
| H04 | *Hprt1* | 0.2595 |
| H05 | *Rplp0* | 0.1117 |
| H06 | *Hgdc* | 0.3374 |
| H07 | *Rtc* | 0.4203 |
| H08 | *Rtc* | 0.4826 |
| H09 | *Rtc* | 0.8858 |
| H10 | *Ppc* | 0.3275 |
| H11 | *Ppc* | 0.3076 |
| H12 | *Ppc* | 0.3579 |
